# The Role of Vitamin D in Kidney Transplantation Outcomes: A Systematic Review

**DOI:** 10.3390/life12101664

**Published:** 2022-10-20

**Authors:** Georgios Koimtzis, Leandros Stefanopoulos, Verity Brooker, Georgios Geropoulos, Christopher G. Chalklin, Sapna Gupta, Eliot Carrington-Windo, Maria Papaioannou, Theodosios S. Papavramidis

**Affiliations:** 1 Cardiff Transplant Unit, University Hospital of Wales, Cardiff and Vale University Health Board, Cardiff CF14 4XW, UK; 2 Lab of Computing, Medical Informatics and Biomedical Imaging Technologies, Aristotle University of Thessaloniki, 54124 Thessaloniki, Greece; 3 Department of Academic Surgery, The Royal Marsden Hospital Foundation Trust, 203 Fulham Rd., London SW3 6JJ, UK; 4 Laboratory of Biological Chemistry, Faculty of Health Sciences, School of Medicine, Aristotle University of Thessaloniki, 54124 Thessaloniki, Greece; 5 1st Propaedeutic Surgical Department, University Hospital of Thessaloniki AHEPA, Aristotle University of Thessaloniki (AUTH), 1st St. Kiriakidi Street, 54621 Thessaloniki, Greece

**Keywords:** vitamin D, kidney transplant, systematic review

## Abstract

The aim of this systematic review is to assess the impact of vitamin D on the outcomes of kidney transplantation and investigate whether its deficiency is associated with a negative impact. **Methods:** We conducted a systematic literature search in PubMed, Scopus and Cochrane databases, as well as gray literature. Ultimately, 16 articles with an average of 255.75 patients were included in this review. These articles compared the long-term outcomes of vitamin D deficiency and/or vitamin D supplementation therapy on kidney transplant recipients by assessing various parameters. **Results:** Most of the included studies showed a negative effect of vitamin D deficiency on kidney transplantation by being associated with a worse graft function, higher incidence of acute rejection episodes, higher incidence of proteinuria and lower overall graft and patient survival rate. **Conclusions:** We suggest that patients awaiting kidney transplantation have a careful evaluation in order to assess their vitamin D status and the optimal supplementation therapy. Regular follow-up of vitamin D levels post-transplant is also suggested. Prospective studies will be needed to establish the positive effects of vitamin D supplementation therapy on kidney transplant outcomes.

## 1. Introduction

Kidney transplant (KT) has been recognized as the optimal method of treatment for patients with end stage kidney disease (ESKD) from the early 1990s as it offers a survival advantage compared to the alternative of dialysis [1]. In recent years, short term outcomes in transplantation have been improved, with 1-year patient and graft survival rates exceeding 90%, but maintaining long-term survival still remains challenging [2]. Kidney graft survival is evaluated according to the duration of time with a functioning graft and preservation of graft function, while minimizing the modifiable risk factors is the main target to improve graft outcomes [3]. Several immunological and non-immunological factors have been related to kidney graft deterioration [4]. Significantly better outcomes have been associated with lower donor and recipient age at the time of transplantation, shorter duration on dialysis, lower panel of circulating reactive antibodies, absence of acute rejection episodes and three or less HLA mismatches [3].

Traditionally, vitamin D is known to play a major role in bone homeostasis and calcium and phosphate metabolism [5]. However, recently it came to light that vitamin D has the ability to modulate the innate and adaptive immune responses since immune cells, such as T-cells, B-cells, monocytes, macrophages and dendritic cells express vitamin D receptors on their surface, while they also have the ability to synthesize its active metabolite [5,6]. In vitro studies have also shown that vitamin D promotes a more tolerogenic immunological status [7]. Additionally, low levels of serum 25-hydroxyvitamin D have been associated with an increased risk of developing various immune-related diseases and disorders such as psoriasis, type 1 diabetes mellitus, multiple sclerosis, rheumatoid arthritis, tuberculosis, sepsis, inflammatory bowel disease, respiratory tract infections, and more recently, COVID-19 infection [8]. The signaling pathway of vitamin D is modulated by the vitamin D receptor (VDR), a specific zinc-finger nuclear receptor [9]. The functions of vitamin D are sub-categorized into genomic and non-genomic. The former is mediated through the VDR transcriptional effects inside the cell nucleus, while the latter by rapid signaling originating from the cell membrane and/or cytoplasm. Emerging evidence highly suggests that vitamin D enhances immunity by providing protection against pathogens, while, at the same time, it poses an immunosuppressive role by preventing the detrimental effects of prolonged inflammatory responses to the host [9].

Vitamin D is also known to possess renoprotective properties [10,11]. It has negative feedback on the renin–angiotensin–aldosterone system (RAAS) and can reduce RAAS-induced renal fibrogenesis [12]. Moreover, animal models have shown that vitamin D supplementation can decrease proteinuria, a recognized risk factor for progressive renal failure, as a result of its anti-proliferative effects [13]. Human studies have also shown that calcitriol and paricalcitol supplementation are associated with significant reduction in proteinuria in cases of IgA and diabetic nephropathy [14,15]. Based on these findings, RAAS inhibition and proteinuria reduction are two potential mechanisms by which vitamin D could have a positive effect on kidney graft function.

The aim of this systematic review is to assess the impact of vitamin D as an immune response modulator and renoprotective agent on the outcomes of KT and investigate whether its deficiency is associated with a negative impact.

## 2. Methods

This study is a systematic review that was performed without a pre-existing registered protocol. It includes studies on the role of vitamin D on kidney graft survival rate published in the English literature between January 2001 and July 2021. Older studies were considered outdated and therefore were not included in the final analysis. Animal studies, studies on pediatric patients, case reports and other systematic reviews were also excluded. A thorough and systematic electronic literature search was conducted in PubMed, Scopus and Cochrane databases. Additional searches for gray literature were conducted on the websites of international transplant, renal and endocrine societies as well as the records of relevant conferences. The search was carried out using the following search string of MeSH keywords:

(((vitamin d[Title/Abstract]) OR (vitamin d[MeSH Terms])) AND ((((kidney transplantation[Title/Abstract]) OR (renal transplantation[Title/Abstract])) OR (kidney graft[Title/Abstract])) OR (renal graft[Title/Abstract]))) AND (((((survival rate[Title/Abstract]) OR (outcome[Title/Abstract])) OR (prognosis[Title/Abstract])) OR (survival[Title/Abstract])) OR (survival rate[MeSH Terms]))

At first, articles were screened based on their title and duplicate records were removed. Then records’ eligibility was evaluated based on their title and abstract and further exclusion took place for articles that were considered irrelevant. Finally, full-text articles were screened for eligibility based on whether relevant data on patients’ vitamin D status and transplant related outcomes were available and the remaining ones were selected for qualitative analysis. Each article was afterwards studied independently for data extraction. Data extracted, included the number of patients in each study, patients’ demographics (age and sex), study design, vitamin D status and outcomes. Literature search and assessment of articles of eligibility for inclusion in the study was performed by two independent reviewers (GK and LS). In cases of disagreement between reviewers, a third independent reviewer (CC) was involved and, ultimately, either a consensus was reached, or the majority opinion was used for the analysis.

This study was prepared according to the PRISMA checklist.

## 3. Results

**Figure 1:**
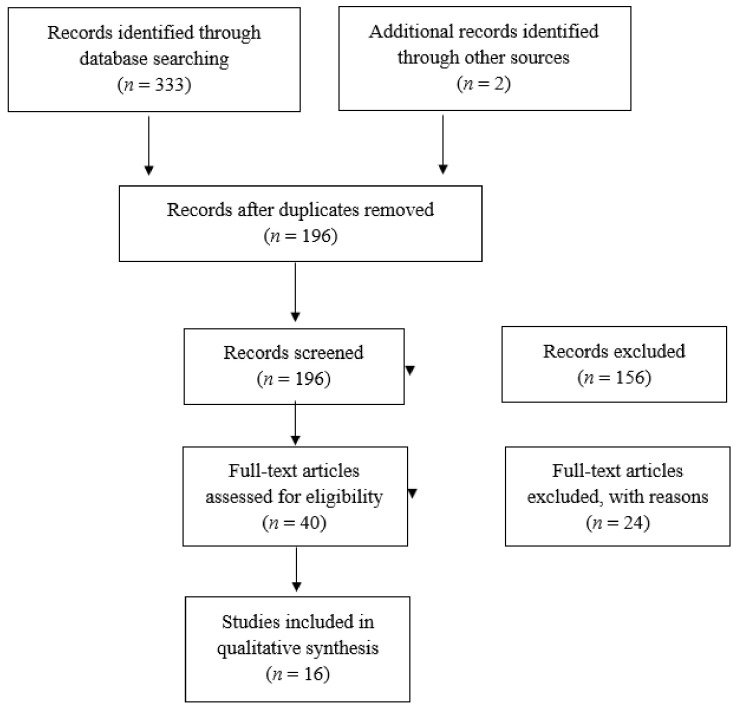
Flowchart depicting the selection process for inclusion of manuscripts in the article.

The initial search on the electronic databases yielded 333 articles, while two more were revealed by searching grey literature. After removal of duplicates, 196 articles remained that were screened for relevance by title and abstract. Out of these 196 articles 40 were eligible for full-text analysis. Ultimately, sixteen articles were included in this review [16,17,18,19,20,21,22,23,24,25,26,27,28,29,30,31] (Figure 1). The rest of the articles were excluded as the design, protocol and/or methodology used investigated a different PICO question to those of our study. Most of the studies (ten) included in the review were prospective/cohort studies, four of them were case-control studies and the remaining two were cross-sectional studies. The number of patients ranged from 52 to 762 (average 255.75) (Table 1). Among the studies that were included in this review, there was a large discrepancy regarding the assessment of vitamin D status and the definition of deficiency. Four studies [24,25,29,30] included patients that received vitamin D supplementation therapy (interventional group) and two of them [25,29] did not measure vitamin D levels to distinguish between sufficient and deficient patients. Eight studies [19,21,22,23,24,26,27,30] divided patients into two groups (sufficient and deficient) using various cutoff values and timing of measuring vitamin D levels. Five studies [16,17,18,20,28] divided patients into three groups (deficient, insufficient and sufficient) while one study [31] further subdivided insufficiency into mild and moderate. Patient follow-up also ranged between one and ten years. 

Various parameters were also used to associate vitamin D levels with positive or negative outcomes of renal transplant. These mainly included the estimated glomerular filtration rate (eGFR) and serum creatinine to assess graft function, episodes of acute rejection, proteinuria, viral infections, overall graft survival and mortality (Table 2). Nine studies [16,18,19,20,21,23,24,26,30] showed a negative effect of vitamin D deficiency in patients’ eGFR and/or serum creatinine at various time cut-offs. Four studies [19,22,28,30] showed a higher incidence of episodes of acute rejection in patients with vitamin D deficiency while only one [17] showed no association between the two. Moreover, four studies [18,26,27,31] showed that patients with vitamin D deficiency had significantly higher rates of proteinuria. Regarding post-transplant CMV and BK virus infections, one study [30] showed lower incidence in patients with normal levels of vitamin D, while another one [22] showed no difference in the infection rate between sufficient and deficient patients. Finally, three studies [21,25,28] showed better patient and/or graft survival in cases of vitamin D sufficiency, while one study [23] showed no correlation between vitamin D status and patient or graft survival.

The measurement of eGFR was used in Mehrota et al. [16] and showed there was a trend supporting better eGFR at 1 year in patients who were not vitamin D deficient. The study also supported the fact that often at the time of transplant, patients are not adequately replaced with vitamin D and subsequently are potentially not being optimized before transplant [16]. Rosina et al. [18] followed a similar vein looking at the association between vitamin D status and eGFR and proteinuria. Their study was cross-sectional in design and defined vitamin D deficiency as a serum 25-hydroxyvitamin D (25(OH)D) level of <16 ng/mL and insufficiency as 16–30 ng/mL [18]. Their study also supports the notion that there is a strong association between low vitamin D levels in renal transplant recipients and decreased eGFR and increased proteinuria [18].

Mehrota et al. [19] evaluated 57 patients in their cohort study, measuring vitamin D levels 3 months before and 3 months after renal transplant. They compared this with measurements of the patients’ eGFR at regular intervals post-transplant. In comparison with Rosina et al. [18], their definition of deficiency of vitamin D was a level of <20 ng/mL which certainly raises the need for a universal definition of ‘deficiency’ and ‘insufficiency’ to ensure some kind of uniformity across studies. Key findings of Mehrota et al. [19] found that 54.4% of renal transplant recipients were classed as vitamin D deficient after transplant, and that this correlated with a ‘significantly lower eGFR and higher serum creatinine values’ [19] than their vitamin D sufficient counterparts. It was also noted that acute rejection was more prevalent within the deficient subgroup [19], which raises the need for further studies to ensure a longer and healthier lifespan for transplanted kidneys.

Following on from highlighting acute rejection episodes, Obi et al. [20] used a measure of number of treatments with IV methylprednisolone to understand the role between vitamin D concentrations and subsequent function of the transplanted kidney. They also measured eGFR as an indicator of kidney function. Again, they had a varying definition of what constituted deficiency, inadequacy and sufficiency, making it difficult to compare studies, however they did find a causal association between patients who were not sufficient in vitamin D and eGFR decline at less than a decade after transplant [20]. Additionally, the requirement for IV methylprednisolone to treat acute rejection episodes was much more likely within the patient group with a poor vitamin D status [20], highlighting further the knock-on effects of inadequate supplementation of vitamin D. Obi et al. [20] do emphasize that they could not study the effect of replacing vitamin D as none of their patients received therapy, and suggested further studies to explore this theory.

Keyzer et al. [21] go further to suggest it is not just the functioning of the transplanted graft that is associated with vitamin D levels, but the all-cause mortality of the patient themselves. This independent association was demonstrated in their observational study, consisting of 435 renal transplant patients [21]. Their definition of poor vitamin D status was of a 25(OH)D level of <12 ng/mL and found that this was independently associated with both a swift eGFR reduction as well as a higher risk of all-cause mortality [21].

Lee et al. [22] were able to go further with comparative variables than Obi et al. [20] as they were able to study patients who were supplemented with vitamin D after transplant and measure the incidence of acute cellular rejection. A total of 61.5% of their patient group were defined as deficient (<20 ng/mL) and of these patients there was a higher incidence of transplant rejection than that of their sufficiently replaced counterparts [22]. Furthermore, it was found that if patients received adequate replacement within 3 months of receiving their graft, there was a much lower risk of rejection as opposed to not being fully supplemented [22]. The study does note that the type of supplementation received was not uniform across the patient groups, and this could have potentially affected the likelihood of rejection due to slightly differing immunological impacts [22].

Bienaime et al. [23] produced a prospective study on kidney transplant outcomes and their levels of vitamin D for 634 patients. The main outcome of the study was that although low 25-hydroxyvitamin D (<15 ng/mL) at 3 months did not correlate with graft failure, it was associated with a reduction in measured GFR+/− standard deviation after 1 year (55 +/− 17 mL/min as opposed to 59 +/− 17 mL/min for >15 ng/mL levels at 3 months) [23]. Again, this finding supports the notion that vitamin D plays a crucial role within the immune response of the recipient and subsequently the level of function of the graft. Bienaime et al. [23] do highlight that some limitations within their study, particularly with reference to the group that were treated for their inadequate vitamin D levels; there was not enough follow up to produce any meaningful data on the effects of replacement, unlike Lee et al. [22].

One study which does focuses particularly on the role of supplementation with 1,25- dihydroxyvitamin- D3 in the context of renal allograft function, is that of O’Herrin et al. [29]. Their focus was upon understanding how the use of oral calcitriol on transplant patients affected the creatinine and graft survival length, with particular focus upon those with ‘chronic allograft nephropathy’ [29]. Renal function was assessed through pre and post calcitriol therapy and was analyzed through a means of ‘general linear mixed modeling’ [29] to obtain a value set as the ‘slope of serum creatinine’ [29]. What the study found was that after starting calcitriol, there was a marked decrease in the rate of reduction in graft function [29]. Additionally, the grafts themselves were functioning for longer in those with treatment, as opposed to those with no calcitriol supplementation [29]. What was more important, was the fact that there were no adverse side effects noted with the use of the supplement [29], which only adds to the potential benefits of certain supplements to support functioning renal transplants. What they do highlight, is the need for further investigation into the mechanisms behind the results, similar to Lee et al. [27].

Moscarelli et al. [30] take a more detailed approach to not only the administration of calcitriol, but how 1, 25 dihydroxyvitamin-D3 levels affect rejection episodes and CMV infection rates in renal transplant patients. They delve further into the correlation between biopsy proven rejection and if calcitriol use affects these outcomes. They found clear results which showed that 1,25- dihydroxyvitamin- D3 deficiency and no supplementary calcitriol were independent risk factors for acute rejection proven with biopsy, as well as CMV and BK virus infection [30]. There was also a strong correlation between the overall functioning of the graft, with improved renal function for those who had adequate levels, as well as using calcitriol [30]. Another highlight of the study was the observation that only two users of calcitriol lost their graft, in comparison to 11 who were part of the control group [30]. This again illustrates the potentially huge benefits to ensuring adequate vitamin D levels within transplant patients, if there is this much a difference in graft loss between those who receive supplements, and those who do not. A particular strength of this study is that it combines not only proving acute rejection with biopsy alongside an association with 1,25-dihydroxyvitamin-D3 deficiency but also that it proves a possible treatment for this deficiency in the use of calcitriol [30].

A study of 90 post- transplant patients by Falkiewicz et al. [24] focused on the effects of low 1,25-dihydroxyvitamin D upon graft outcomes. Specifically, the paper identified that there was a higher incidence of delayed graft function within the subset of patients who were identified as deficient [24]. Unlike other studies mentioned within this review, all of the patients were receiving alfacalcidol supplementation before the transplant, 83% of which were deemed to be severely 1,25-dihydroxyvitamin D deficient immediately after receiving the graft [24]. It was only after measuring the concentrations on a monthly basis that the correlation between these poor levels and increased risk of delayed graft function was noticed. Additionally poorer outcomes in general were noted within this group, including five failed grafts and four cardiovascular-related deaths [24].

Ozdemir et al. [25] specifically compare the outcomes of two separate groups of post- transplant patients, 40 of which were treated with vitamin D, whilst the remaining 62 were not. This study took the hypothesis to a new level by, amongst other parameters, studying the infiltration of macrophages within peritubular capillaries and the interstitium [25]. Their aim was to understand how the status of vitamin D within the patients affected the likelihood of rejection within their transplants, as well as understanding the microscopic complications of untreated vitamin D deficiency [25]. The research found that there was a 26% increased difference between the loss of the graft at 29.2 +/− 15 months in the non- supplemented group as opposed to the supplemented group at 43.2 +/− 13 months [25]. They also found a much lower infiltration rate of macrophages within the interstitial and pertitubular capillaries in patients who were adequately replaced with vitamin D, as well as reduced destruction of peritubular capillaries [25]. The study confidently states that there is a definitive ‘positive impact on long term graft survival’ [25] with adequately replaced vitamin D patients, explaining that the mechanism is likely due to the reduction in renal HLA-DR expression through its immunosuppressive properties.

Another study which focusses on the anti-inflammatory properties of vitamin D and renal transplant function is one published in 2009 by Sezer et al. [26]. Their aim was to measure renal function via serum creatinine levels, albumin, C-reactive protein and urinary protein at 1 year post transplant. They divided groups based upon if their serum vitamin D levels were either below or above 20 micrograms/ L-groups I and II, respectively [26]. At 1 year follow up, it was noticed that group I showed evidence of a much higher creatinine than those of their group II counterparts, additionally they had higher proteinuria [26]. One limitation of the study was that to the best of our knowledge, they did not mention if the group with adequate vitamin D status was supplemented, or if this was a natural occurrence. It could have provided more evidence to support potential plans to implement a pre transplant plan to supplement patients with vitamin D to improve outcomes, if we were to know if and what the patients with adequate vitamin D status were taking to achieve this level.

25-hydroxyvitamin D levels divided patients within Lee et al.’s study [27] into poor status (<30 ng/mL) and adequate status (>30 ng/mL). Again, there is shown to be much disparity between the definition of inadequacy, as if we were to compare to Sezer et al.’s study [26], many of their ‘adequate’ patients would be deemed to be inadequate in Lee et al.’s [27] parameters; making their findings difficult to interpret. Either the higher limitations are unnecessarily extreme, if Sezer et al. still found positive correlation between lower levels and poorer outcomes, or we need to find out if better outcomes are ensured in a logarithmic fashion—exponentially getting better the higher the vitamin D status. Lee et al. [27] also use proteinuria as a measure for kidney function upon transplant patients, similar to that of Sezer et al. [26], finding that there was an association between increased proteinuria and vitamin D ‘insufficiency’ [27]. Unlike Sezer et al. [26] they highlight the need for further research into the exact mechanism of vitamin D supplementation and reduction in proteinuria [27].

Another study focusing upon proteinuria is Filipov et al.’s [31], which included a total of 230 kidney transplant recipients. The study itself consisted of taking 25-hydroxyvitamin D levels alongside a sample of urine to test for protein during a routine follow up appointments [31] They excluded patients who had received the transplant within 12 months, those with ‘unstable graft function’ [31] and those who had previously had a parathyroidectomy [31]. What was noted within the study, was the speculation that vitamin D had specifically renoprotective properties, particularly within this study’s focus upon the effect on proteinuria. What was found was a ‘significant negative relation[s]’ [31] between the functioning of the graft and 25-hydroxyvitamin D concentrations. In addition, there was a strong positive association between proteinuria and rejection episodes [31]. What is noted, is that there is no clear mechanism of action described for these results within this study, however they do highlight the need for further insight into the potential renoprotective qualities vitamin D supplements may possess [31].

A prospective observational cohort study undertaken in Denmark by Thorsen et al. [28] followed a total of 762 patients with their first renal transplant. They undertook testing to understand if vitamin D status was associated with particular outcomes [28]. In particular they focused on patients who had graft failure or patients who subsequently died [28]. The study included transplant recipients from between 2007 and 2012 who were then tested for vitamin D status 10 weeks after receiving their graft [28]. The median follow up was just under 7 years. Interesting results were produced, in particular the fact that 86% of patients with adequate vitamin D levels had a functioning transplant after 5 years, compared with only 79% of those with ‘deficiency’[28] and 76% of those with ‘insufficiency’ [28], thus highlighting further the strong evidence towards ensuring patients are correctly identified and treated for vitamin D insufficiency, not only for the short-term outcomes, but potentially more importantly for the long-term results [28].

## 4. Discussion

To the best of our knowledge this is the first systematic review that summarizes the evidence of the long-term influence of vitamin D deficiency on the outcomes of kidney transplantation. A recent meta-analysis of 28 studies on the same subject by Yin et al. [32] showed that vitamin D deficiency is common following KT and is associated with a high risk of mortality and adverse outcomes including infection and acute rejection. However, this study focused only on early vitamin D deficiency and assessed kidney graft function only one year post-operatively. Additionally, studies that investigated the role of vitamin D supplementation therapy were excluded from this meta-analysis. Another recent meta-analysis of 14 studies by Mirzakhani et al. focused only on the role of vitamin D on acute rejection following KT and revealed an 82% higher chance of acute rejection in patients suffering from vitamin D deficiency [33]. 

Based on our review, most of the available studies indicate that vitamin D deficiency is associated with adverse outcomes following kidney transplantation and especially higher mortality rate, worse renal function (lower eGFR and higher creatinine), higher rate of proteinuria and higher incidence of acute rejection. Moreover, vitamin D supplementation therapy seems to ameliorate these adverse outcomes.

In addition, our review revealed that vitamin D status is neither assessed universally by using a common time and level cut-off nor is it evaluated routinely in every KT recipient. Since secondary hyperparathyroidism is a common complication of chronic kidney disease with complex bone, mineral, metabolic and organic disorders [34] that is accompanied by dysregulation of vitamin D metabolism [35], we suggest that every patient with ESKD awaiting a renal transplant has an early assessment of their vitamin D status. Additionally, there should be an evaluation by a multidisciplinary team of transplant nephrologists and endocrinologists, as well as specialized dietitians to assess the need for supplementation therapy and the most optimal way to achieve a normal vitamin D status. Also, we suggest that these patients, following a successful KT, should have their vitamin D levels measured regularly as part of their follow-up. It is important to underline that general agreement on the optimal target of vitamin D level to be achieved, the doses that should be used, and how long the supplementation should be maintained, has not been reached yet [36].

Vitamin D deficiency is a common problem following solid organ transplantation [37]. Strict sun protection is recommended to transplant recipients to minimize the risk of skin cancer. However, this approach increases the risk of severe vitamin D deficiency, as its primary source is endogenous production in the skin through the action of the sun [38]. Hence, it is of paramount importance and relevance to transplant recipients to detect and manage vitamin D deficiency considering its immunomodulatory and renoprotective properties. Also, a study on 289 KT recipients by Filipov et al. [39] revealed that vitamin D status is negatively affected by calcineurin inhibitors (e.g., tacrolimus), which nowadays are the most commonly used type of immunosuppressant. However, vitamin D status was not affected using newer mTOR inhibitors (sirolimus, everolimus). Therefore, appropriate management of immunosuppression therapy in KT recipients who also suffer from vitamin D deficiency mandates a more careful and individualized approach. Moreover, a study on 461 KT recipients by van Ballegooijen et al. [40] indicated that combined vitamin D and K deficiency is associated with a higher risk of premature all-cause mortality and death-censored graft failure. The same study indicated that KT recipients on vitamin D treatment suffering from vitamin K deficiency had a higher risk of premature mortality and graft failure compared to patients who were not on vitamin D supplements. This indicates even further the complexity of vitamin D status’ effect on KT outcomes and thus stresses the importance of a cautious and highly specialized approach. Finally, a few observational studies have confirmed the association of vitamin D deficiency and increased risk of cardiovascular disease, infection and cancer in KT recipients [41]. Since these are the most common causes of death following KT, we further stress the importance of identifying the optimal range of vitamin D supplementation that could provide KT recipients with the most clinical benefits.

Finally, a key issue that also needs to be mentioned is that most of the included studies only prove the association between low levels of vitamin D and adverse outcomes in kidney transplant patients, without proving causality. As a matter of fact, recurrence of the primary disease or a de novo disease in the kidney graft could lead to proteinuria, worse kidney function and mortality. It is already well-known that the cumulative incidence of allograft glomerulonephritis is 42% at 10 years post-transplantation and is responsible for 18–22% of death-censored graft failure [42]. Also, idiopathic focal segmental glomerulosclerosis (FSGS) is known to recur in almost 30% of the cases following kidney transplantation. Moreover, membranous nephropathy recurs in 40–50% of cases following transplantation and leads to graft failure in 10–15% after 10 years [42]. Membranoproliferative glomerulonephritis has a similar graft loss rate at 10 years post transplantation due to recurrence. As a result, it is evident that low vitamin D levels could be attributed to recurrence of the primary disease or a de novo allograft nephropathy, which are the actual causes of a poor outcome. However, measuring the vitamin D level in kidney transplant patients still remains important as it could serve as a warning sign of allograft nephropathy.

This systematic review has several strengths and limitations. Firstly, it includes a wide variety of studies that used different methods of assessing vitamin D status, different time cutoffs and different follow-ups. Therefore, statistical analysis of these outcomes was not performed as the great heterogeneity would lead to inconclusive or unreliable results. Secondly, only ten of the included studies were prospective and all of them were observational. However, most of them revealed that vitamin D deficiency is associated with inferior outcomes in KT recipients.

## 5. Conclusions

Vitamin D deficiency is associated with adverse short- and long-term outcomes following KT, including worse graft function, episodes of acute rejection, proteinuria, viral infections, overall graft survival and patients’ mortality. However, the clinical effects of the deficiency as well as the supplementation remain largely unknown, hence the need for prospective studies to be carried out to confirm the adjuvant effect of vitamin D supplementation therapy.

## Figures and Tables

**Table 1:** Characteristics of each study included in the review.

Study	Number of Patients	Age (Mean)	Male/Female	Study Design
Mehrotra et al. [16]	52	34.85 ± 9.95	43/9	Cohort
Zimmerman et al. [17]	327	51 ± 14	67% male	Cohort
Rosina et al. [18]	195	47·6 ± 11.2	114/81	Cross-sectional
Mehrotra et al. [19]	57	34.28 ± 11.12	50/7	Cohort
Obi et al. [20]	264	49.0 ± 12.3	61.3% male	Cohort
Keyzer et al. [21]	435	52 ± 12	51% male	Cohort
Lee et al. [22]	351	52.5 ± 14.3 (control) 52.1 ± 13.1 (case)	221/130	Case-control
Bienaimé et al. [23]	634	48.3 ± 13.4	372/262	Cohort
Falkiewicz et al. [24]	90	42.7 ± 11.4	53/37	Cohort
Özdemir et al. [25]	102	29.1 ± 11.1 (control) 28.3 ± 11.3 (treatment)	68/34	Case-control
Sezer et al. [26]	64	38.61 ± 1.05	38/26	Cohort
Lee et al. [27]	95	48 (median)	54/41	Cross-sectional
Thorsen et al. [28]	762	57	515/247	Cohort
O’Herrin et al. [29]	76	41.3 ± 11.8 (treatment) 44.5 ± 13.2 (control)	45/31	Case-control
Moscarelli et al. [30]	360	51	252/108	Case-control
Filipov et al. [31]	230	43.00 ± 12.68	148/82	Cohort

**Table 2:** Outcomes of included studies.

Study	Use of Vitamin D	Outcomes and Notes
Mehrotra et al. [16]	25 (OH) vitamin D levels of >30 ng/mL were considered sufficient and <20 ng/mL were deficient while 20–30 ng/mL were the insufficient vitamin D status	Comparison between groups with e-GFR <60 and ≥60 mL/min/1.73 m^2^ showed that post-transplant eGFR at 12 months correlated with vitamin D sufficiency.
Zimmerman et al. [17]	Patients were classified as vitamin D sufficient, insufficient and deficient at the time of transplant. Follow-up for one year.	Serum concentration of 25-hydroxyvitamin D and 1,25-dihydroxyvitamin D were not associated with acute rejection.
Rosina et al. [18]	Vitamin D deficiency <16 ng/mL, insufficiency 16–30 ng/mL and sufficiency >30 ng/mL	Lower serum levels of vitamin D were associated with increasing proteinuria and decreasing eGFR.
Mehrotra et al. [19]	Patients divided into two groups <20 ng/mL and >20 ng/mL vitamin D levels at 3-months post-transplant	Patients with vitamin D deficiency at 3-months post-transplant had lower eGFR and higher serum creatinine levels at 3-month, 6-month and 1-year post-transplantation. Patients with vitamin D deficiency had more episodes of acute rejections. Patients with post-transplant vitamin D levels at 3 months of >30 ng/mL had the best graft function at one year post transplant.
Obi et al. [20]	Vitamin D deficiency <12 ng/mL, insufficiency 12–20 ng/mL, sufficiency ≥ 20 ng/mL	Vitamin D inadequacy and deficiency were independently associated with rapid eGFR decline at less than 10 years after transplantation (*p* < 0.05).
Keyzer et al. [21]	Vitamin D deficiency defined as level <12 ng/mL	Vitamin D deficiency significantly associated with all-cause mortality and higher annual change in eGFR.
Lee et al. [22]	Vitamin D deficiency defined as levels less than 20 ng/mL Vitamin D therapy initiated within the first 90 days of transplantation in 133 of 216 vitamin D deficient patients.	Patients with vitamin D deficiency were at greater risk of developing acute cellular rejection (ACR) within the first year of transplantation (*p* = 0.03). Hazard ratio for ACR increased from 3.3 (*p* = 0.02) observed in the entire group of vitamin D deficient patients to 4.4 (*p* = 0.004) in the subset of vitamin D deficient not treated with 1,25(OH)D3 and the hazard ratio decreased to 1.5 (*p* = 0.52) in the subset of vitamin D deficient treated with 1,25(OH)D3. No difference in the incidence of CMV disease and BK virus associated nephropathy during the first year of transplantation between the vitamin D deficient group and the sufficient group. No difference in the eGFR at 12 months post transplantation between the two groups.
Bienaimé et al. [23]	Vitamin D deficiency was defined as 25[OH]D < 15 ng/mL 3 months post transplantation	1.Similar risk of death in patients with low and high 3-month 25(OH)D and 3-month 1,25(OH)D. 2. No association between graft loss and 3-month 25(OH)D or 3-month1,25(OH)D levels, (*p* = 0.65) 3. 12-month 25(OH)D concentration was not correlated with 12-month mGFR (r = 0.04; *p* = 0.43). 4. 3-month 25(OH)D concentration was independently associated (*p* = 0.01) with interstitial fibrosis (IF) and tubular atrophy (TA) progression, but the increased risk of IF/TA progression was not associated with an increased incidence of biopsy-proven acute rejection.
Falkiewicz et al. [24]	All patients received vitamin D supplementation before transplantation. Deficiency defined as level of 1,25-dihydroxyvitamin D concentration below 15 pg/mL. Follow-up for two years.	Higher incidence of delayed graft function (*p* = 0.01) in patients with deficiency and negative correlation between 1,25-dihydroxyvitamin D and serum creatinine concentration.
Özdemir et al. [25]	Patients divided in two groups based on administration or not of vitamin D (calcitriol) therapy	Five-year graft survival was significantly and positively affected by vitamin D therapy (*p* < 0.001).
Sezer et al. [26]	Vitamin D level measured. Deficiency defined as level below 20 μg/L. Follow-up for one year.	Patients in the deficient group showed significantly higher creatinine (*p* < 0.001) and proteinuria levels (*p* < 0.05) than those in the non-deficient group
Lee et al. [27]	Vitamin D insufficiency defined as level ≤ 30 ng/mL (or 75 nmol/L)	A significantly higher prevalence of proteinuria was observed in the vitamin D insufficient group (*p* = 0.02)
Thorsen et al. [28]	Vitamin D deficiency defined as serum 25(OH)D concentrations <30 nmol/L (<12 ng/mL), insufficiency 30–50 nmol/L (12–20 ng/mL), and sufficiency >50 nmol/L (>20 ng/mL). Follow-up for one year.	Frequency of rejections was highest in the group with low vitamin D. Patient and graft survival were significantly better in patients with vitamin D sufficiency at 10 weeks post-transplant compared with patients with vitamin D insufficiency or deficiency.
O’Herrin et al. [29]	Patients in the intervention group (n = 26) received treatment with calcitriol. Follow-up was for >1 year	Calcitriol treatment was associated with a significant improvement in graft survival compared to no calcitriol exposure (*p* < 0.03)
Moscarelli et al. [30]	121 patients treated with calcitriol therapy. Vitamin D deficiency as circulating levels less than 20 pg/mL.	Incidence of biopsy proven acute rejection, CMV infection and BK virus infection were significantly lower in patients using supplements. Calcitriol treatment was associated with a significant improvement in graft function compared to no calcitriol exposure.
Filipov et al. [31]	Vitamin D status divided in four categories: Sufficient ≥ 75 nmol/L Mild insufficiency 50–75 nmol/L Severe insufficiency 25–50 nmol/L Deficiency < 25 nmol/L	Negative coefficient between vitamin D status and proteinuria.

## Data Availability

All data generated or analyzed during this study are included in this published article.
